# Mechanism of pyroptosis in neurodegenerative diseases and its therapeutic potential by traditional Chinese medicine

**DOI:** 10.3389/fphar.2023.1122104

**Published:** 2023-01-13

**Authors:** Yanfang Liao, Xue Wang, Liting Huang, Hu Qian, Wei Liu

**Affiliations:** ^1^ Science and Technology Innovation Center, Guangzhou University of Chinese Medicine, Guangzhou, Guangdong, China; ^2^ Department of Breast Cancer Oncology, Foshan No 1 Hospital, Foshan, China; ^3^ The First Clinical Medicine College of Guangzhou University of Chinese Medicine, Guangzhou, Guangdong, China; ^4^ Integrative Cancer Centre, The First Affiliated Hospital of Guangzhou, University of Chinese Medicine, Guangzhou, China

**Keywords:** pyroptosis, neurodegenerative diseases, traditional Chinese medicine, neuroinflammation, neuroprotection

## Abstract

Neurodegenerative diseases (NDs) are disorders characterized by degenerative degeneration of neurons and loss of their function. NDs have a complicated pathophysiology, of which neuroinflammation and neuronal death are significant factors. The inflammatory process known as pyroptosis (“fiery death”) is caused by a family of pore-forming proteins called Gasdermins (GSDMs), which appears downstream from the activation of the inflammasome. Clear evidence of enhanced pyroptosis-related proteins activity in common NDs has coincided with abnormal aggregation of pathological proteins (such as Aβ, tau, α-synuclein et al.), making pyroptosis an attractive direction for the recent study of NDs. The purpose of this review is to provide an overview of the molecular mechanisms driving pyroptosis, the mechanistic links between pyroptosis and NDs, and emerging therapeutic strategies in Traditional Chinese Medicine (TCM) to inhibit pyroptosis for the treatment of NDs.

## Introduction

Currently, with the dramatic rise in the aging population, the growing incidence of neurodegenerative diseases (NDs) is a ticking time bomb, which could become one of the biggest healthcare economic challenges we face today (Eleftheriadou et al., 2020). Neurodegeneration refers to the complex process of progressive degeneration or abnormal death of neurons, resulting in a series of incurable and debilitating diseases (Heemels, 2016). From a pathological perspective, neuronal loss associated with gliomas, protein misfolding and deposition, resulting in abnormal filamentous deposits is the main symptom of NDs (Fang et al., 2020; Ikram et al., 2020). There has been evidence that neuronal degeneration is related to pyroptosis, a form of inflammatory programmed cell death (Moujalled et al., 2021). It is believed that GSDMs are intracellular proteins involved in the process of pyroptosis. GSDMs can be proteolyzed by certain proteases (caspase and granzyme) to form pore-forming domains (GSDMs-N) and repressor domains (GSDMs-C), where release proinflammatory molecules as GSDMs-N form pores on plasma membranes, which leads to osmotic cell lysis and increases inflammation (Tsuchiya, 2021). Inflammasomes serve as the most important upstream signals of pyroptosis pathway, and inhibition of inflammasome activation can reduce GSDMD expression and pyroptosis in neurons, thereby playing a neuroprotective role (Tan et al., 2014). For instance, NLRP3 deficiency improves spatial memory impairment in AD model by reducing the generation of caspase-1 and IL-1β, promoting the clearance of Aβ (Heneka et al., 2013). Therefore, treatments that target proteins associated with the pyroptosis process may deliver new treatment options for NDs. Traditional Chinese medicine (TCM), which has been used for more than 2000 years, has been shown to improve neurodegenerative symptoms such as cognitive impairment, disorientation, and loss of consciousness (Cai et al., 2018). Treatments with TCM for neurodegenerative diseases have been found to have a remarkable effect, and there are increasing studies on the effects of TCM on pyroptosis in the treatment of NDs. Thus, it is vital for pathological research, clinical prevention, and drug development to investigate how TCM affects NDs-associated pyroptosis.

## Pyroptosis

### The brief introdution of pyroptosis

Researchers discovered that the death of mouse macrophages or human monocytes brought on by *Shigella flexneri or Salmonella* infection was not “apoptosis,” but rather a caspase-1 dependent programmed necrosis, as early as the late 1990s (Zychlinsky et al., 1992; Cookson and Brennan, 2001). In order to describe inflammatory programmed necrosis, the term “pyroptosis” was created to characterise inflammatory programmed necrosis by fusing the Greek terms “pyro” and “ptosis,” which refer to fever and falling (Shi et al., 2015). Pyroptosis was formerly thought to be monocyte death caused by caspase-1 (Bergsbaken et al., 2009). After some time, Shi et al. discovered that pyroptosis may also result from the activation of caspase-4/5/11 to cleavage GSDMD protein, which is composed of two domains (GSDMD-C and GSDMD-N), with the GSDMD-N having an ability to oligomerize, causing the cell membrane to rupture, releasing inflammatory substances (Shi et al., 2014). Pyroptosis is therefore defined as programmed necrosis occurring within the gasdermin.

### Gasdermin, the executioner of pyroptosis

The family of proteins known as GSDMs has a wide range of functional properties and is expressed in many cell types and tissues. The paralogous genes for gasdermin A (GSDMA), gasdermin B (GSDMB), gasdermin C (GSDMC), gasdermin D (GSDMD), gasdermin E (GSDME, also known as DFNA5), and Pejvakin (PJVK, also known as DFNB59) are found in human, while the paralogous genes for GSDMA1-3, GSDMC1-4, GSDMD, DFNA5, and DFNB59 are found in mice (He et al., 2015; Ding et al., 2016). As a structural feature, all GSDM proteins, except for DFNB59, have pore-forming N-terminal domains, autoinhibitory C-terminal domains, and loop domains between N- and C-terminal (Kuang et al., 2017). When these two domains are proteolytically cleaved, the intramolecular inhibitor of the cytotoxic domain is released, which enables it to insert into cell membranes and form large oligomeric pores that disrupt ionic homeostasis and induce cell death (Aglietti and Dueber, 2017).

### The characteristics of pyroptosis

In morphology, pyroptosis has both partial characteristics of apoptosis, including Annexin V staining, DNA breakage, condensation of chromatin and so on (Kurokawa and Kornbluth, 2009). However, pyroptosis is mediated by the activation of a family of caspases by extracellular or intracellular stimuli (e.g., bacteria, viruses, toxins, and chemotherapeutic drugs), which ultimately leads to the release of inflammatory factors (like IL-1β and IL-18) to amplify the inflammatory response. Early pyroptosis was attributed to caspase-1-dependent death (Broz and Dixit, 2016). Later, it has been found that caspases 4/5/11 activate GSDMD by proteolysis, leading to the development of plasma membrane holes and pyroptosis (Kayagaki et al., 2015; Shi et al., 2015). Additionally, caspase-3 (Wang et al., 2018) and caspase-8 (Orning et al., 2018) both have the ability to trigger pyroptosis by cleaving GSDME and GSDMD, respectively. It is therefore clear that GSDMs, which go over to the plasma membrane, where they oligomerize and build pores, are responsible for the execution of pyroptosis (Shi et al., 2017). There is evidence that pores created by GSDMs-N, such as GSDMD-N, have a larger inner diameter and may facilitate the passage of IL-1β and IL-18 (Ding et al., 2016), which are involved in a wide variety of cellular processes, including inflammation, proliferation, and differentiation (Rostovtseva and Bezrukov, 1998; Lim et al., 2014; Ding et al., 2016). Adenosine triphosphate (ATP), high mobility group protein 1 (HMGB1), and lactate dehydrogenase (LDH) are among the unmodified damage-associated molecular patterns (DAMPs) released during plasma membrane rupture (McKenzie et al., 2020). It is also possible to infiltrate Ca^2+^ from the extracellular environment during pyroptosis when GSDMD holes are created in the plasma membrane, which activates calcium citrate and forms the endosomal sorting complex necessary for transport (ESCRT) (Ruhl et al., 2018; Davis et al., 2019). By encouraging budding out and excision of damaged membranes, the ESCRT machinery, in contrast, facilitates plasma membrane healing (Ruhl et al., 2018). Therefore, the balance between the amount of GSDMD pores and Ca^2+^-dependent regulatory mechanisms may decide, at least in part, whether GSDMD pores trend to pyroptosis or plasma membrane repair when it is triggered.

### Canonical pathway and non-canonical pathway

**FIGURE 1:**
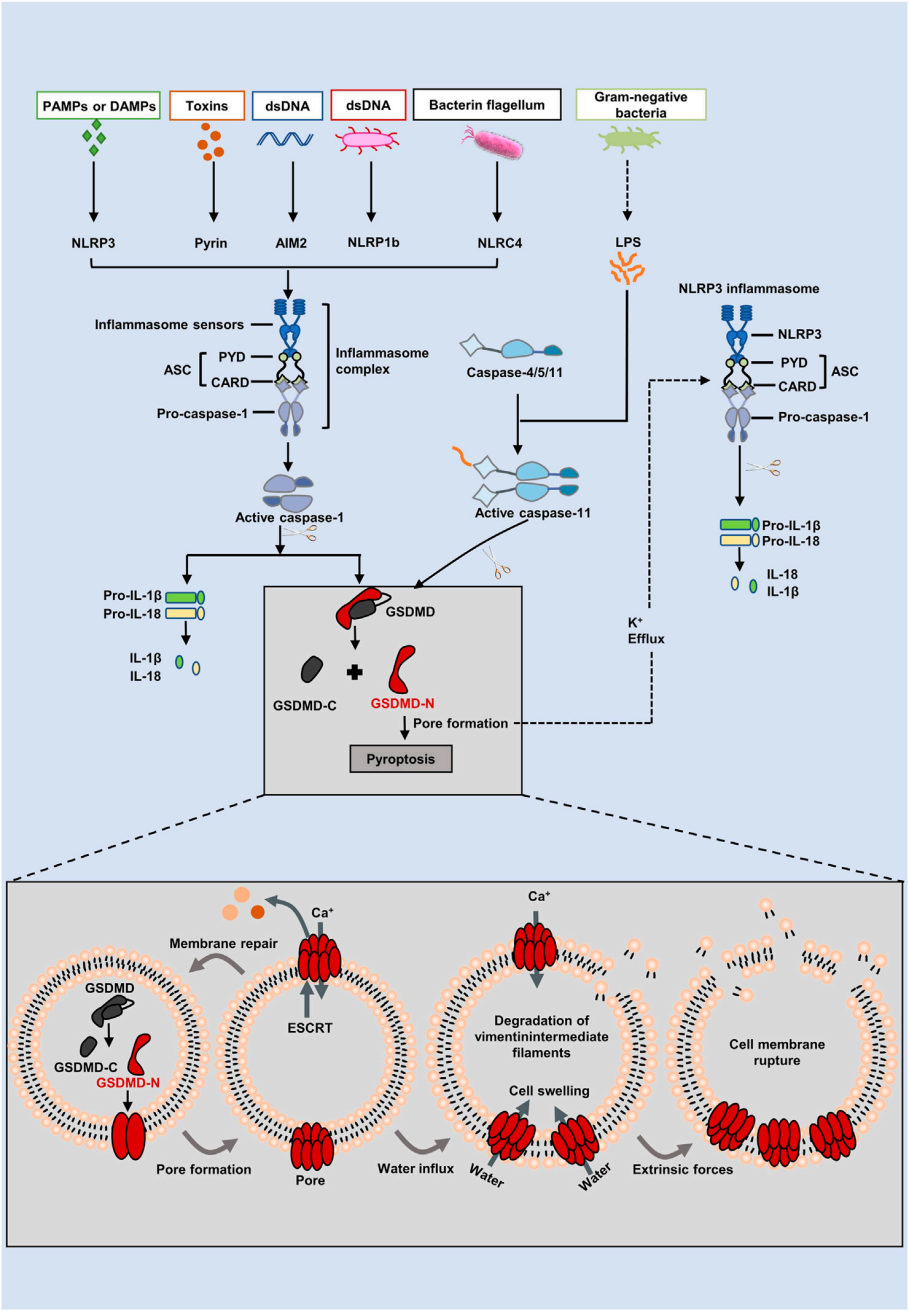
The canonical and non-canonical inflammasome pathways of the pyroptosis. In the canonical pathway, intracellular signaling molecules stimulate PAMPs and DAMPs, which activates caspase-1 and causes it to cleave GSDMD into GSDMD-N. GSDMD-N generates holes resulting in cell swelling, inflammatory mediators release, and pyroptosis. Inflammatory caspases are triggered in the non-canonical pathway by the caspase-4/5/11 binding to intracellular lipopolysaccharide (LPS). GSDMD is broken down into GSDMD-N by activated caspases, which binds to lipids in the plasma membrane to produce huge oligomeric holes that allow the discharge of cellular contents and cause cell death. Furthermore, GSDMD cleavage results in K^+^ efflux, which eventually facilitates the NLRP3 inflammasome assembed, IL-1β and IL-18 cleavaged.

Pyroptotic death is typically mediated by inflammasome assembly that is accompanied by GSDMD cleavage and IL-1β and IL-18 release (Xia et al., 2019). PAMPs (pathogen associated molecular patterns) or DAMPs (unaltered damage-associated molecular patterns) cause multiprotein complexes called inflammasomes to form (Malik and Kanneganti, 2017). As an initial step in the assembly of inflammasomes, pattern recognition receptors (PRRs), also known as inflammasome sensors, are located in the cytoplasm and are capable of recognizing PAMPs and DAMPs (Liston and Masters, 2017). When cells are stimulated by signaling molecules such as bacteria and viruses, PRR attaches to pro-caspase-1 and ASC to create inflammasomes. As a result of stimulation by bacteria and viruses, PRR joins to pro-caspase-1 and ASC to establish inflammasomes (Place and Kanneganti, 2018). Currently, nucleotide-binding oligomerization domain-like receptors (NLRs), such as NLRP1, NLRP3, and NLRC4, absent in melanoma 2 (AIM2), and pyrin are now the most prevalent PRRs that may construct classical inflammasoms (Broz and Dixit, 2016). Generally, most inflammasomesare are comprised of there main types: leucine rich repeat containing proteins (NOD-like receptors (NLRs), apoptosis associated speck-like protein containing a caspase-recruitment domain (ASCs) and pro-caspase-1 (Zheng et al., 2020). Nucleotide-binding and oligomerization domains (NACHT) characterized by carboxy-terminal leucine-rich repeats (LRRs) and CARD or pyrin domains (PYD) are characteristic of these NLRs (Lamkanfi, 2011; Zitvogel et al., 2012). Depending on whether they have a PYD or CARD at the N-terminus, NLRs are either NLRPs or NLRCs. The N-terminal region of NLRCs may include one or more CARDs, while the N-terminal region of NLRPs includes PYD (Lamkanfi and Dixit, 2012; Lamkanfi and Dixit, 2014). With NLRP1, Anthrax lethal toxin, muramyl dipeptide, and Toxoplasma gondii have been studied more extensively (Mitchell et al., 2019). It is possible to activate NLRP3 by PAMPs (including bacteria, viruses, and fungi) or DAMPs (such as reactive oxygen species (ROS), ATP, and endogenous damage signals) (Elliott and Sutterwala, 2015). The NLRCC4 receptor recognizes flagellin from bacteria and type 3 secretion components (Zhao et al., 2011). Additionally, AIM2 and pyrin can also form inflammasomes. The DNA-binding HIN-200 domain and PYD domain of AIM2 detect double-stranded DNA from bacteria or viruses (Hornung et al., 2009). There are three domains in pyrin: two B-boxes, a PYD domain, and a SPRY/PRY domain at the C-terminus. A major function of pyrin is to recognize inactivating modulators that are mediated by bacteria’s toxins or effectors in the inactivation of Rho guanosine triphosphatases (Ratner et al., 2016). As a consequence of the activation of PRRs, pro-caspase-1 is subsequently cleaved into caspase-1. On the one hand, GSDMD is split into its C- and N-terminus by caspase-1, and the GSDMD-N punctures the cell membrane quickly and repeatedly to cause pyroptosis (Sborgi et al., 2016). On the other hand, caspase-1 is also responsible for cleaving pro-IL-1β/18, which ultimately brings about the release of mature forms of IL-1β/18 *via* pores formed by GSDMD and ultimately drives pyroptosis (He et al., 2015). The diagram for this pathway can be found in Figure 1.

The activation of pro-caspase-11 in mice (pro-caspase-4/5 in humans) is one way that the non-canonical inflammasome pathway, which operates independently of the traditional inflammasome complex, may be generated (Shi et al., 2017) (Figure 1). Here, mice caspase-11 (human orthologs caspase-4/5) is activated by binding of the N-terminal CARD to lipopolysaccharide (LPS) identified in Gram negative bacteria (Chu et al., 2018). Upon activation of caspase-4/5/11, GSDMD is then effectively cleaved into GSDMD-N, and therefore pyroptosis is brought out (Aglietti et al., 2016). A note of interest, pro-IL-1β and pro-IL-18 cannot be cleaved by caspase-4/5/11. GSDMD, however, triggered K^+^ efflux and induced NLRP3 inflammasome assembly, which promoted IL-1β and IL-18 maturation and secretion *via* the NLRP3/caspase-1 pathway after being cleaved by caspase-4/5/11(Shi et al., 2017). In addition, through the pannexin-1/ATP pathway, caspase-11 activates P2X7 channels, causing damage to the membrane and pyroptosis (Yang et al., 2015).

### Other molecules mediated pathway

**FIGURE 2:**
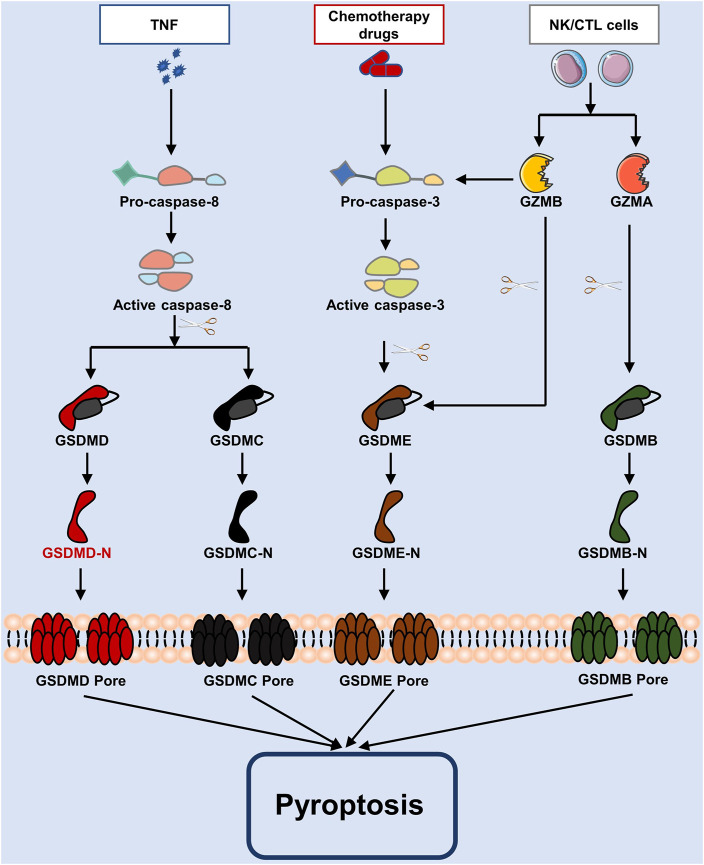
The apoptotic caspase and granzymes-mediated pyroptosis process. Caspase-3 and caspase-8 are activated in pyroptosis by chemotherapy drugs and TNF, whereas caspase-3/GSDME, caspase-8/GSDMD, caspase-8/GSDMC, etc., are involved pathways. As well, in cytotoxic lymphocytes, GZMA or GZMB are inserted *via* perforin, and GSDMB or GSDME are cleaved to cause pyroptosis.

GSDMs may also be triggered by caspase-3/8, which results in cell death, in addition to inflammatory caspase-1/4/5/11. There are several recent studies have found that chemotherapy treatments cause caspase-3 to become active. Once this caspase-3 is activated, at Asp270, it breaks down GSDME to create GSDME-N, which travels to the plasma membrane and promotes the formation of pores (Wang et al., 2017; Rogers et al., 2017). In contrast, pyroptosis is inactivated when GSDMD is cleaved at Asp87 by caspase-3 (Taabazuing et al., 2017). In a *murine macrophage Yersinia* infection study, caspase-8 was to activate GSDMD downstream of transforming growth factor (TGF) β-activated kinase-1 (TAK1) and thereby trigger pyroptosis (Sarhan et al., 2018). Also, activated caspase-8 is capable of cleaving GSDMC, initiating pyroptosis (Zhang et al., 2021). According to another research, Granzyme A (GZMA) from cytotoxic T lymphocytes cleaves GSDMB, releasing GSDMB-N-terminus to cause pores in the membrane, and further promoting pyroptosis (Zhou et al., 2020). Note that the cleavage of GSDME produces an N-terminal fragment of GADME, which encourages the creation of membrane pores, hence causing pyroptosis, following caspase-3 activation by granzyme B (GZMB) generated from natural killer cells (Liu et al., 2020). These pathways are shown in Figure 2.

### Therapeutic strategies for the inhibition of pyroptosis

Currently, the more widely studied in therapeutic strategies to inhibit pyroptosis mainly include pro-inflammatory caspases inhibitors, NLRP3 inflammasome inhibitors and GSDMD inhibitors. The caspase-1 inhibitor Vx-765, which may pass the blood-brain barrier and has no overt adverse effects, has been extensively utilized in clinical investigations of epilepsy and psoriasis (Flores et al., 2018; Mangan et al., 2018). Recently, cell-permeable synthetic peptides based upon the cleavage site of GSDMD (Ac-FLTD-CMK) was also created to prevent the caspase-1 family of proteases from working (Yang et al., 2018). The most common NLRP3 inhibitor, diarylsulfonylurea compound CP-456733 (CRID/MCC950), has been shown to suppress NLRP3 inflammasome activation brought on by ATP and other stimuli (Mangan et al., 2018). GSDMD inhibitors are still in the developmental stages, and the ones that have received the most attention from researchers include necrosulfonamide (NSA), disulfiram (DSL) and an NF-κB inhibitor (Bay 11-7082). NSA is not specific for GSDMD even though it has been demonstrated to reduce pyroptosis in a sepsis model since it can also prevent the creation of mixed lineage kinase domain-like protein (MLKL) pore-related to necrosis (Sun et al., 2012). DSL and Bay 11-7082 may prevent pyroptosis caused by GSDMD. Additionally, it has been shown that disulfiram may save mice with sepsis and an MS mouse model from death (Li et al., 2019).

## NDs and pyroptosis

**FIGURE 3:**
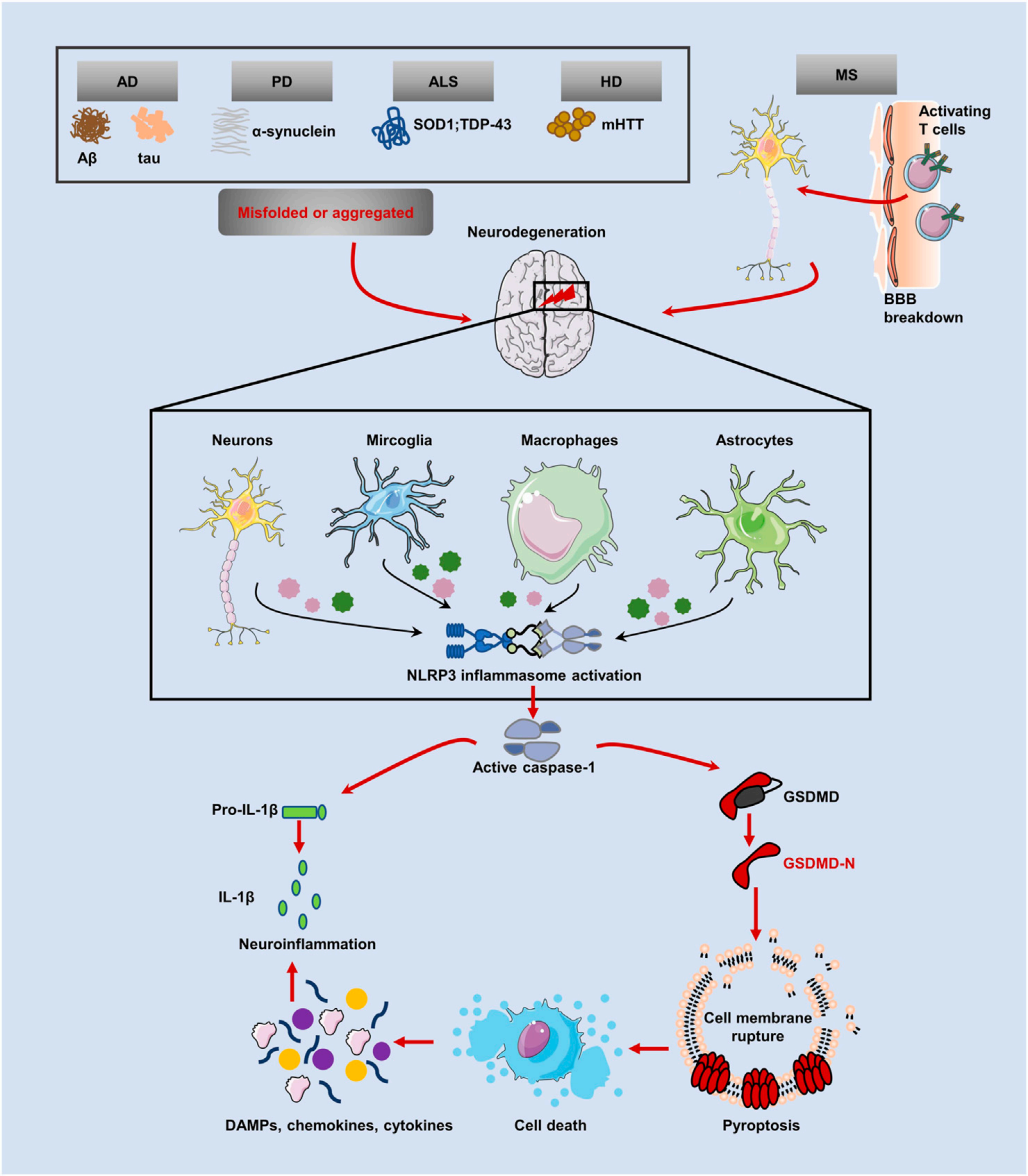
Relationship between pyroptosis and NDs. Inflammasomes can be activated in several NDs, including AD, PD, ALS and HD, with protein misfolding and abnormal aggregation (accumulation of amyloid Aβ, hyperphosphorylated tau protein and *a*-synuclein aggregates) and autoimmune mediated neurodegenerative injury (MS). NLRP3 inflammasome cleavaged GSDMD and pro-IL-1β by caspase-1, causing pyroptosis and subsequent release of mature cytokines, amplifying the neuroinflammatory response.

The term “neurodegenerative disease” is used to describe a range of disorders resulting from myelin degeneration or loss of neurons. We are experiencing a dramatic increase in NDs patients due to the rapid aging of our society (Katsuno et al., 2018). Protein aggregation, immunological dysregulation, and aberrant cell death are all features of NDs, which eventually cause brain or peripheral nervous system gradually loses neurons (Katsnelson et al., 2016). Pyroptosis, an inflammatory programmed form of death, has been linked to the emergence of NDs including AD, PD, HD, ALS, and MS, according to research. Several pyroptotic inflammasome components, including NLRP3, caspase-1, IL-1β and GSDMD have been reported in NDs (Table 1). Figure 3 provides a schematic representation of the relationship between neurodegenerative diseases and pyroptosis.

**TABLE 1:** Evidence for different mechanisms of pyroptosis in neurodegeneration diseases.

Neurodegeneration disease	Pathogebic proteins	Mechanism	The PMID of references
AD	Aβ	Activating the expression of NLRP1, caspase-1, GSDMD	25144717; 31111399
AD	Aβ	NLRP3, caspase-1, and GSDMD are activated	32521573; 23254930
			34435574; 32892233
AD	Aβ	Activated NLRP3, caspase-1 and IL-1β	23831373; 18604209
AD	Aβ, ASC- Aβ	ASC- Aβ complex activates NLRP3, caspase-1, GSDMD	32187546
AD	tau	NLRP3/IL-1β induced tau protein hyperphosphorylation	31748742; 33667787 31731189
AD	tau	Inhibition of caspase-1 inhibited tau hyperphosphorylation and pyroptosis	31731189
AD	—	Exhibiting more NLRP1, caspase-1, IL-1β, and GSDMD in AD brains	26939933; 17658666
			33587329
PD	α-syn	Activating the expression of NLRP3, IL-1β	23383169; 31036561
			32795556; 30594776
PD	—	NLRP3-caspase-1-GSDMD activation in PD animal model	35143076; 30381407
			28247334; 34780806
PD	—	Exhibiting more NLRP3, caspase-1, ASC, and IL-18 in PD patients’ plasma, PBMCs, and substantia nigra	30381407; 31915018
			33398042
ALS	SOD1, TDP-43	Caspase-1, IL-1, IL-18, and NLRP3/ASC activation	26200799; 28936769
			27957680; 29575052
			20616033; 31596526
			35227277
ALS	—	Activating NLRP3 expression and GSDMD antibody reactivity	35227277; 35867112
			34883532
HD	HTT	Activating NLRP3, caspase-1, and IL-1 expression in R6/2 mice	32821438; 33066292
			35219323; 31375685
HD	—	The HD patients activated NLRP3, caspase-1, and IL-18	10353249; 18625748
			26297319
MS	MOG	Inflammasomes (NLRP3, caspase-1, IL-1β) were activated in MS model	28583987; 31997770
			27997058; 21106820
MS	MOG	Activatng GSDMD in EAE model of MS.	29895691; 31467036
MS	—	The MS patients’ NLRP3, caspase-1, IL-1β, and GSDMD levels increase	29895691; 15471361
			19162335; 24657029

※Abbreviations: AD, Alzheimer’s disease; PD, Parkinson’s disease; ALS, amyotrophic lateral sclerosis; HD, Huntington’s disease; MS, multiple sclerosis; Aβ, amyloid beta; α-syn: α-synuclein; SOD1, Cu2+/Zn2+ superoxide dismutase; TDP-43, TAR DNA binding protein 43; HTT, huntingtin protein; MOG, myelin oligodendrocyte glycoprotein.

### Pathological link between pyroptosis and NDs

Pathological features most common to NDs are protein misfolding and abnormal aggregation in neurons (Hipp et al., 2014). Amyloid beta (Aβ) and hyperphosphorylated tau protein-coated neurofibrillary tangles, which are the hallmarks of AD, a chronic ND (O’Brien and Wong, 2011). Caspase-1 could be activated by inflammasomes such NLRP1, NLRP3, NLRC4, AIM2, and pyrin to cause pyroptosis (Man et al., 2017). It has been shown that Aβ aggregation might trigger the NLRP1 inflammasome to cause pyroptosis mediated by caspase-1 and the release of IL-1β *via* the P2X7-purinoctor/pannexin1 signaling pathway (Yap et al., 2019). Aβ could directly produce neuronal pyroptosis that is reliant on NLRP1 and caspase-1 in cultured neurons of the cortical region. In contrast, suppressing NLRP1 in APPswe/PS1dE9 mice brought out a significantly reduction in the amount of neuronal pyroptosis as well as cognitive impairment (Tan et al., 2014). It is interesting to note that during the progression of AD, microglial pyroptosis encourages the formation of the ASC-Aβ Complex, which not only exacerbate Aβ formation of oligomers and aggregates, but also caused the formation of the NLRP3 inflammasome, caspase-1 activation, IL-1β maturation and GSDMD cleavage, and promoted pyroptosis in nearby microglia (Heneka et al., 2018; Luciunaite et al., 2020). Similarly, Aβ_1–42_ induces pyroptosis in cortical neurons through NLRP3-caspase-1-dependent pyroptosis as well (Han et al., 2020). Additionally, NLRP3 inflammasomes may cause tau pathology in AD since they act as upstream signals of tau (Ising et al., 2019). Li Y et al. found that caspase-1 silencing could attenuate cognitive dysfunction and neuronal damage in a mouse model of cerebral tau hyperphosphorylation mimicked by intracerebroventricular injection (forskolin, FSK and streptozotocin, STZ); Notably, lithium chloride (LiCl) treatment not only inhibited tau hyperphosphorylation but also decreased production of caspase-1, IL-1β, and IL-18 and prevented pyroptosis (Li et al., 2020). Additionally, tau protein triggers the NLRP3-ASC axis, which in turn causes inflammasome activation and microglial pyroptosis (Stancu et al., 2019). The concepteal platform of pyroptosis have promoted important efforts to identify plasma-borne indicators of inflammasome markers. Specifically, according to research by Rui et al., the pathophysiology of people with amnestic mild cognitive impairment (aMCL) and Alzheimer’s disease (AD) is strongly associated to the proinflammatory cytokine IL-1β. Moreover, peripheral blood mononuclear cells (PBMCs) of aMCL and AD patients showed canonical inflammasome signaling and GSDMD-induced pyroptosis activation (Rui et al., 2021).

As a typical neurodegenerative disorder, PD caused by the abnormal loss of dopamine (DA) neurons in the substantia nigra area and the production of Lewy bodies (Braak et al., 2003). In neurons, *a*-synuclein (α-syn) aggregates generate Lewy bodies, impairing DA neuron function and eventually leading to neuronal death (Serpell et al., 2000). Several early studies have shown that there is a role for neuroinflammation in the pathogenesis of PD (Wang et al., 2015). In this situation, a toll-like receptor on the cell membrane recognizes aggregated α-syn and activates the NF-κB pathway, thereby promoting the production of precursor proteins for IL-1β (Bauernfeind et al., 2009). After that, α-syn aggregation has been claimed to cause IL-1β (Codolo et al., 2013) and ASC (de Alba, 2019) production *via* NLRP3 inflammasome activation in monocytes/macrophages and microglia. Caspase-1 is recruited and activated by NLRP3 inflammasome activation to enhance IL-1β maturation and the triggering of pyroptosis, which in turn stimulates the release of IL-1β, which further destroys dopamine neurons (Block et al., 2007). Moreover, NLRP3 or caspase-1 deletion proved to result in reduced microglial activation, prevented DA neuron loss, and motor deficits were repaired in the N-methyl-4-phenyl-1,2,3,6-tetrahydropyridine (MPTP)-induced PD model (Qiao et al., 2017; Lee et al., 2019). Another study illustrated, α-synuclein activates NLRP3 in LPS and α-synuclein-stimulated mouse microglia, leading to IL-1β and ASC release but does not mediate pyrolysis (Gordon et al., 2018). It is possible that synuclein concentrations may be quantified in future studies to determine whether synuclein ultimately induces pyroptosis. Of note, PD patients’ substantia nigra densa expressed a higher abundance of activated inflammasomes and overexpressed ASC (Gordon et al., 2018), as well as higher NLRP3 expression in PBMCs and IL-18 levels in plasma compared with healthy controls (Fan et al., 2020). Although these reports do not definitively demonstate that α-syn ultimately induces pyroptosis, this may correlate with α-syn concentration and metabolism. However, another study demonstrated that pussian blue nanozyme (PBzyme) inhibited neuroinflammation and reduced dopaminergic degeneration by mediating microglial pyroptosis *via* ROS/NLRP3/caspase-1/GSDMD pathway (Ma et al., 2022). These studies suggest that inhibition of pyroptosis or pyroptosis related pathway proteins may alleviate PD progression.

ASL is a persistent loss of spinal motor neurons with patients experiencing progressive motor dysfunction as a ND (Swanton et al., 2018). Protein aggregates containing Cu^2+^/Zn^2+^ superoxide dismutase (SOD1) (Rosen et al., 1993) and TAR DNA binding protein 43 (TDP-43) (Neumann et al., 2006) are the main pathologiccal features of ASL. A recent work discovered that monocyte-derived cells from ASL patients with TPD-43 inclusions were significantly more likely to activate the NLRP3 inflammasome than cells from healthy controls (Quek et al., 2022). Furthermore, patients with ALS who are affected by the NLRP3 inflammasome are less resilient to non-motor symptoms due to the assembly of this inflammasome (Banerjee et al., 2022). Several other reports have also explained that NLRP3/ASC oligomerization and IL-1β secretion have been attributed to SOD1 and TDP-43 protein aggregation (Gordon et al., 2018; Voet et al., 2019). The canonical NLRP3 inflammasome activates pyroptosis of neurons in the ventral horn of the lumbar spinal cord in ALS mice, amplifying neuroinflammation (Zhang et al., 2022). Inflammasomes such NLRC4, AIM2, and caspase-1 have also been shown to be elevated in the SOD1-G93A mouse model, in addition to NLRP3, ASC, and IL-1β (Deora et al., 2020). Later studies have shown that in SOD1 transgenic mice, the deletion of caspase-1 or IL-1β delayed the onset of illness (Meissner et al., 2010). In a clinical study performed on patients with ALS, GSDMD antibody reactivity was demonstrated in the motor cortex and white matter, as well as increased NLRP3 expression in microglia (Van Schoor et al., 2022). Although these studies implicated pyroptosis as a driver of ALS, their mechanisms are still unclear.

The pathogenic basis of HD is the aggregation of the huntingtin protein (HTT), which results in the formation of inclusion bodies (Ha and Fung, 2012). There are distinct neuroinflammatory features at the sites of brain lesions in HD patients, according to the current study (Bjorkqvist et al., 2008). Inflammasome components of the NLRP3 are expressed in both the central and peripheral nervous system according to clinical and preclinical study, for example, HD patients’ PBMCs express higher levels of NLRP3 than healthy controls (Glinsky, 2008). HD patients have been shown in many studies to have elevated amounts of the IL-1β in their plasma, as well as in the striatum and cerebral cortex (Bjorkqvist et al., 2008; Politis et al., 2015). When compared with wild-type mice, the expression of NLRP3 and active-caspase-1 was higher in the striatum of transgenic R6/2 mutant mice (Paldino et al., 2020). In the meanwhile, researchers have shown that HD patients’ cerebral cortexes have greater amounts of mature IL-1β than healthy control individuals’ do. As well as this, administration of a caspase inhibitor (zVAD-fmk) in R6/2 mice has also been shown to prevent the development of motor dysfunction (Ona et al., 1999). These results provide credence to the idea that NLRP3 inflammasome activation and the subsequent pyroptosis play a role in HD (Paldino et al., 2020).

As prototypical neuroinflammatory disease, MS is triggered by an immune response that damages the myelin sheath of neurons in the central nervous system (CNS) (Frohman et al., 2006). A model of MS based on the autoimmune encephalomyelitis (EAE) has demonstrated that myelin oligodendrocyte glycoprotein (MOG) increases NLRP3 mRNA levels (Gris et al., 2010). NLRP3 mediates capase-1-activated cytokines, which in turn influence the pathological process of EAE (Gris et al., 2010). Moreover, EAE mice also exhibited GSDMD immunoreactivity and pyroptosis in myeloid cells in the CNS (Li et al., 2019). Similarly, clinical studies also found that PBMCs isolated from MS patients had increased caspase-1 and IL-18 levels (Huang et al., 2004). Additionally, MS patients have higher levels of IL-1β in both plasma and cerebrospinal fluid (CSF) (Dujmovic et al., 2009), and IL-1β levels are directly correlated with the degree of demyelination in the brain (Seppi et al., 2014). Among cadavers from MS patients with frontal white matter, McKenzie et al. detected abundant GSDMD-positive cells (McKenzie et al., 2018). The studies suggest that pyroptosis and MS may be related.

## Effects of traditional Chinese medicine or extracts

**FIGURE 4:**
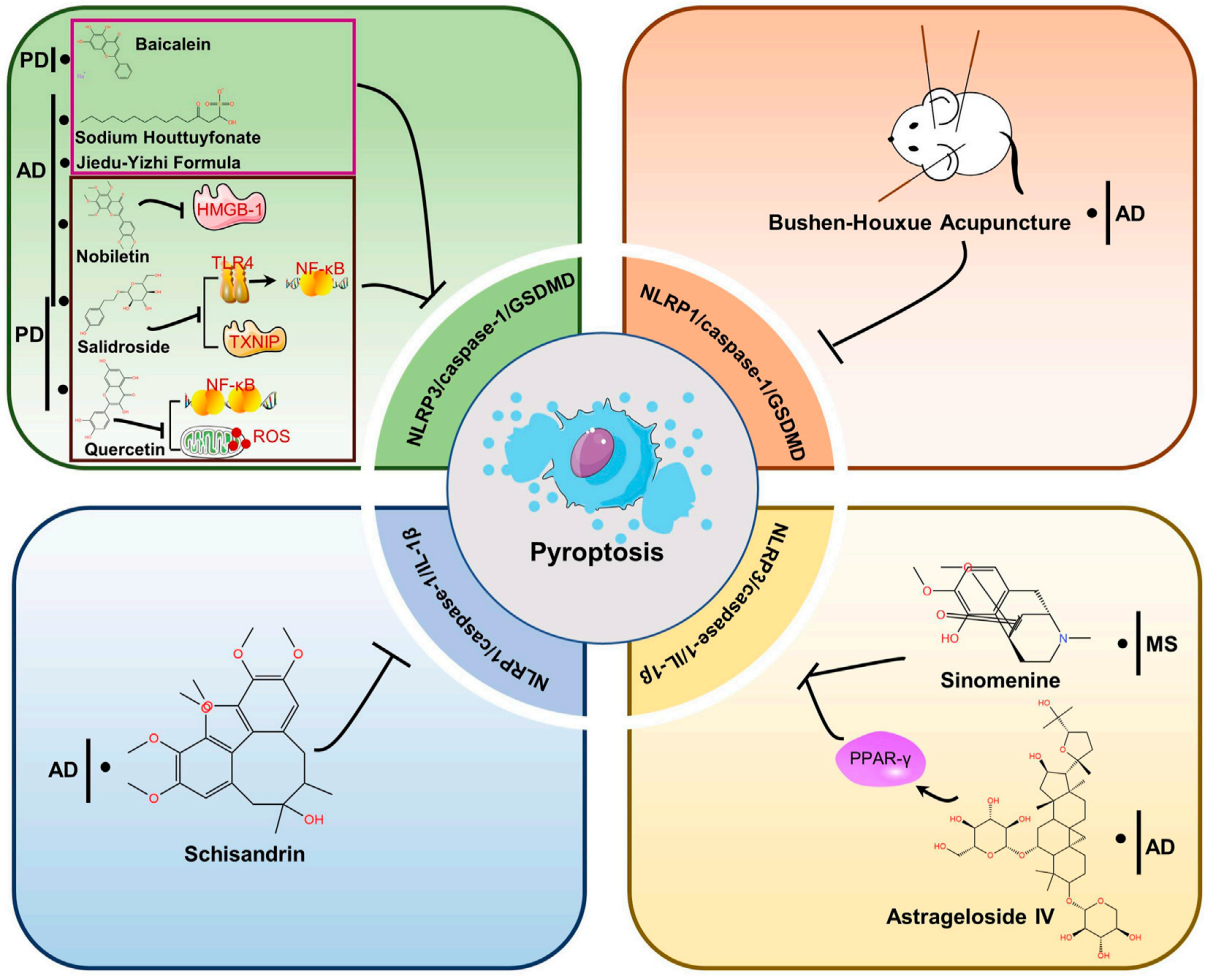
Therapeutic strategies of TCMs for treating NDs by targeting pyroptosis. Baicalein, Sodium Houttuyfonate and Jiedu-Yizhi Formula attenuated neuronal pyroptosis by inhibiting NLRP3/caspase-1/GSDMD pathway. By reducing the expression of HMGB-1, Nobbiletin suppresses NLRP3 and diminishes pyroptosis. Salidroside inhibited either the TLR4/NF-κB pathway or the expression of TXNIP and downregulated the expression of NLRP3, caspase-1 and GSDMD, thus inhibiting pyroptosis to restore neuronal function. Quercetin prevents the generation of NF-κB and ROS, which prevents NLRP3 from being activated. The NLRP1/caspase-1/GSDMD pathway is blocked by Bushen-Houxue Acupuncture, which prevents pyroptosis from occurring. Schisandrin prevents pyroptosis by blocking the NLRP1-caspase-1-IL-1β axis. Sinomenine and Astrageloside IV, however, inhibit the NLRP3-caspase-1-GSDMD axis.

Herbal medicines, including traditional Chinese medicine (TCM), have been utilized for centuries to prevent, treat and cure various diseases (including neurodegenerative diseases) (Yang et al., 2019). With the increasing use of advanced technologies in analytical and biological fields, the powerful and long-lasting therapeutic effects of TCM have been confirmed (Liu et al., 2015). The function of pyroptosis inflammasome pathway in neurodegenerational diseases is being extensively studied. There is increasing evidence that TCMs have neuroprotective effects, and recent studies suggest that the mechanism of action is through inhibition of pyroptosis. In the following sections, we summarized TCM formulas and extracts that exert neuroprotective effects by inhibiting pyroptosis related proteins in NDs (Table 2). As can be seen in Figure 4, the drugs mechanism of action is also depicted in a diagram.

**TABLE 2:** Treatments targeting pyroptosis in TCM for neurodegenerative diseases.

Interventions	Experimental model	Mechanism	The PMID of references
Jiedu-Yizhi formula	A*β*_25-35_-induced AD rats	Blocking the NLRP3-caspase-1-GSDMD and LPS-caspase-1-GSDMD axis to prevent the progression of AD	35341145
Nobiletin	LPS + Nigericin-treated microglia *in vitro*; APP/PS1 mice *in vivo*	Decreasing the expression of HMGB-1 and pyroptosisi-related proteins to mitigating AD	35267136
Astrageloside IV	AβO-induced AD mice	Increasing the expression of PPARγ to inhibiting pyroptosis, neuroinflammation, and tau hyperphosphorylation	34616501
Sodium houttuyfonate	A*β*_1-42_-induced AD mice	Inbibiting the NLRP3/GSDMD pathway to ameliorating A*β*_1-42_-induced memory impairment	34188608
Schisandrin	APP/PS1 mice	Inhibiting of NLRP1 inflammasome-medicated neuronal pyroptosis to inproving cognitive impairment	33542629
Bushen- Huoxue Acupuncture	SAMP8 mice	Inhibiting NLRP1 inflammasome-mediated pyroptosis to attenuating the cognitive defect of AD mice	33574670
Salidroside	A*β*_1-42_-induced AD mice; MPTP-induced PD mice	Inhibiting NLRP3 inflammsome-mediated pyroptosis to ameliorating AD or PD	35126093
			32432571
Quercetin	LPS-induced PD mice	Ameliorating dopaminergic neuron loss by suppressing NLRP3/IL-1β pathway	34082381
Baicalein	MPTP-induced PD mice	NLRP3/caspase-1/GSDMD pathway inhibition reduces neuronal loss and motor dysfunction	32761175
Sinomenine	EAE model mice	Alleviating demyelination and axonal damage by decreasing NLRP3, ASC, and caspase-1 expression	32812186

Pyroptosis in neurodegenerative diseases is primarily mediated by the chassic pathway, which is mediated by the NLRP3 inflammasome. NLRP3 signaling pathway molecules and pyroptosis can be inhibited by TCM or extracts, decreasing the development of NDs like AD, PD, and MS. Jiedu-Yizhi formula (JDYZF) is a TCM formula invigorating kidney and invigorating bone marrow, invigorating phlegm and activating blood flow, and detoxifying blood, which has been invented by Ren Jixue, a master of TCM. This formula has been used in the clinic for many years for the treatment of AD with significant effect. It has been demonstrated that oral administration of JDYZF reversed Aβ_25–35_-induced cognitive impairment, reduced Aβ deposition, and improved neuronal function by inhibiting the expression of NLRP3/caspase-1/GSDMD and LPS/caspase-11/GSDMD axis (Wang et al., 2022). According to Chai et al., nobiletin inhibited neuroinflammation by reducing HMGB-1 and pyroptosis related protein (NLRP3/caspase-1/GSDMD) expression in APP/PS1 mice (Chai et al., 2022). In use for thousands of years, huttuynia cordata has many pharmacological activities, such as anti-inflammatory and anti-viral (Pan et al., 2010). In AD, sodium houttuyfonate (SH), one of the major extracts from houttuynia cordata, has been shown to inhibit NLRP3/GSDMD, a pathway responsible for memory impairment, inflammation, and pyroptosis (Zhao et al., 2021). In addition, astragalus membranaceus, as one of the most commonly used herbs in traditional Chinese medicine, includes components such as polysaccharides, saponins, flavonoids, amino acids and trace elements (Shao et al., 2004; Ragupathi et al., 2008). Astrageloside IV (AS- IV), an active component of astragalus membranaceus, could ameliorate memory impairment and neuronal loss by reducing tau hyperphosphorylation, neuroinflammation and pyroptosis *via* regulating PPARγ (Wang et al., 2021). What’s more, salidroside (Sal), a pharmacologically active component isolated from rhodiola, has also been demonstrated to treat AD (Cai et al., 2021) and PD (Zhang et al., 2020) by preventing pyroptosis mediated by NLRP3 inflammasome. In LPS-induced PD mice, quercetin (Qu) pretreatment ameliorated dopaminergic neuron loss by suppressing microglial activation *via* NLRP3/IL-1β-dependent pathway (Han et al., 2021). Similarly, a flavonoid isolated from scutellaria baicalensis georgi also inhibits NLRP3/caspase-1/GSDMD pathway signaling in PD model, thereby attenuating neuroinflammation, neuronal loss, and motor dysfunction (Rui et al., 2020). Sinomenine, an alkaloid found in the roots of sinomenine, may have potential pharmacological effects against NDs, such as AD and PD, due to its anti-inflammatory and immunosuppressive properties (Tang et al., 2018). A study by Kiasalari and colleagues showed that sinomenine reduced NLRP3, ASC, and caspase-1 levels in spinal cord specimens as well as neuroinflammation, demyelination, and axonal damage (Kiasalari et al., 2021). NLRP1 is also highly conserved in brain spinal neurons and oligodendrocytes, along with NLRP3 (Tan et al., 2014). For example, schisandrin (SCH), as a lignan representing Schisandra chinensis, inhibits the action of the NLRP1 inflammasome on neuronal pyroptosis in AD mice, thereby alleviating cognitive impairment (Li et al., 2021). Additionally, acupuncture is an important therapy of TCM that has been proven to enhance the learning and memory functions of patients with Alzheimer’s (Jia et al., 2017). In their study, Zhang et al. found that bushen huoxue acupuncture prevented NLRP1-mediated pyroptosis and attenuated cognitive decline (Zhang et al., 2021). Compared with AD and PD, the research of TCM-related treatment by pyroptosis pathway in MS, HD and ALS are very few. Further studies are needed.

## Concluding remarks

In this review, we outlined the molecular the mechanisms of pyroptosis. As seen in Figure 3, inflammasomes and pyroptosis can be activated by several molecules that trigger NDs, and inflammatory factors released during pyroptosis can also aggravate neuroinflammation. From the connection between the pathological mechanisms of NDs and pyroptosis, the GSDMD mediated pyroptosis signaling pathway is relatively well defined. Moreover, the pyroptosis pathways mediating neuronal injury in NDs mainly include NLRP3/caspase-1/GSDMD mediated canonical pyroptosis pathway and LPS/caspase-11/GSDMD mediated non-canonical pyroptosis pathway. Insuffciently, the signaling pathways mediated by other proteins from GSDM family are less studied. In fact, the injured hippocampal tissue showed high levels of GSDME expression, which can activate a cytokine storm by triggering an inflammatory response. Here we did not review the reports indicating the pathophysiology of NDs also involves the apoptosis caspases-mediated pyroptosis pathway. What’s more, whether neurological diseases also have molecular target in GZMs-mediated pathway. The use of these targeted molecules for guiding clinical diagnosis and prognosis is of great concern.

Developing drugs will be facilitated by a deeper understanding of the link between pyroptosis and NDs pathogenesis. Numerous studies have confirmed that TCM formulas, extracts, and acupuncture can all improve symptoms of neurodegeneration by suppressing pyroptosis. This creates a role model for the development of more selective TCM medicines for the treatment of NDs. For NDs, these studies are insufficient as they only target NLRP1 or NLRP3/caspase-1/GSDMD canonical inflammasome pathways for suppressing pyroptosis. Furthermore, these drugs inhibit pyroptosis by inhibiting inflammatory factors upstream and downstream of GSDMD, such as NLRP3, caspase-1, and IL-1β. Further research is needed to determine whether GSDM-mediated pyroptosis leads to the development and progression of NDs. These inadequacies make basic research still challenging and we still have a long way to go in order to finally achieve clinical translation.
